# Recombinant bacteriophage LysKB317 endolysin mitigates *Lactobacillus* infection of corn mash fermentations

**DOI:** 10.1186/s13068-020-01795-9

**Published:** 2020-09-08

**Authors:** Shao-Yeh Lu, Kenneth M. Bischoff, Joseph O. Rich, Siqing Liu, Christopher D. Skory

**Affiliations:** 1 Renewable Product Technology Research Unit, National Center for Agricultural Utilization Research, Agricultural Research Service, U.S. Department of Agriculture, 1815 North University Street, Peoria, IL 61604-3902 USA; 2 Agricultural Research Service, U.S. Department of Agriculture, Fort Collins, CO 80526 USA

**Keywords:** Phage endolysin, Contamination, Fuel ethanol, *Lactobacillus*, Antimicrobial

## Abstract

**Background:**

Commercial ethanol fermentation facilities traditionally rely on antibiotics for bacterial contamination control. Here we demonstrate an alternative approach to treat contamination using a novel peptidoglycan hydrolase (LysKB317) isolated from a bacteriophage, EcoSau. This endolysin was specially selected against *Lactobacillus* strains that were isolated as contaminants from a fuel ethanol plant. The LysKB317 gene was recombinantly expressed in *Escherichia coli* as a 33 kDa purified enzyme.

**Results:**

In turbidity reduction assays, the recombinant enzyme was subjected to a panel of 32 bacterial strains and was active against 28 bacterial strains representing 1 species of *Acetobacter*, 8 species of *Lactobacillus*, 1 species of *Pediococcus*, 3 species of *Streptococcus*, and 1 species of *Weissella*. The activity of LysKB317 was optimal around pH 6, but it has broad activity and stability from pH 4.5–7.5 up to at least 48 h. Maximum activity was observed at 50 °C up to at least 72 h. In addition, LysKB317 was stable in 30% ethanol up to at least 72 h. In experimentally infected corn mash fermentations, 1 µM endolysin reduced bacterial load by 3-log fold change, while 0.01 µM reduced bacteria by 2-log fold change. Concentration of fermentation products (ethanol, residual glucose, lactic acid, and acetic acids) for infected cultures treated with ≥ 0.01 µM LysKB317 was similar to uncontaminated controls.

**Conclusion:**

Exogenously added LysKB317 endolysin is functional in conditions typically found in fuel ethanol fermentations tanks and may be developed as an alternative to antibiotics for contamination control during fuel ethanol fermentations.

## Background

Endolysins are peptidoglycan hydrolase enzymes (also known as phage lysins) produced by bacteriophages to enzymatically degrade host bacterium cell wall from within to release progeny virions at the end of lytic multiplication cycle [1]. Due to endolysins’ antibacterial activity, they are considered potential alternatives to antibiotics [2, 3].

The fuel ethanol industry in the United States has experienced a tremendous growth over past decade from 110 plants (6.5 billion gallons per year) in 2007 to 200 plants in 2017 with production capacity approaching 16 billion gallons per year [4, 5]. An estimated production capacity for fuel ethanol would need to reach 60 billion gallons per year by 2030 to meet the proposed US Energy Independence and Security Act (EISA) of 2007, Renewable Fuel Standard (RFS) mandates, and goal set by the environmental protection agency (EPA) and other states to increase higher blend of ethanol in gasoline [6–8]. However, commercial ethanol fuel facilities rarely perform fermentations under aseptic conditions [9, 10]. Fermentation tanks in ethanol production are constantly contaminated with a wide variety of microbes that can cause chronic and acute contaminations in commercial biorefineries [11]. These strains cause both chronic and acute infections in commercial biorefineries and can significantly reduce the level of ethanol production [4, 9, 10, 12, 13]. Potential sources of microbial contamination (bacteria, fungi, and wild yeast) can be found in raw materials such as corn, corn mash, and process water, although through the liquefaction process they appeared to be inactivated [9, 13–15]. Acute contamination often occurs unpredictably and can lead to a costly shutdown of facilities [16]. It is generally believed that lactic acid bacteria (LAB), and predominantly species of *Lactobacillus*, are the primary bacterial contaminants found in fuel ethanol fermentation facilities [17, 18]. In addition to competition of nutrients and substrates with fermenting yeast, bacterial contaminants produced undesirable byproducts such as acetic and lactic acids can inhibit yeast growth [9, 19]. The presence of *Lactobacillus* can cause “stuck fermentations” and decrease yields of ethanol production as *Lactobacillus spp*. compete for resources and negatively impact the health of *Saccharomyces sp*.[4, 16–18, 20]. The solution to combat contamination in the United States has traditionally relied on the usage of antibiotics such as erythromycin, penicillin, and virginiamycin [21]. Concerns over long-term excessive usage of antibiotics are believed to contribute to the emergence of antibiotic resistant bacteria and remains controversial in the ethanol industry [22, 23]. Alternative strategies such as the deployment of all-natural proteinaceous antimicrobial control agents such as endolysin are warranted.

In this study, we described the application of a novel recombinant peptidoglycan hydrolase (endolysin) LysKB317 derived from *Lactobacillus* bacteriophage vB_LfeS_EcoSau (abbreviated as EcoSau); isolated from commercial sauerkraut) to inhibit the growth of lactic acid bacteria known to contaminate ethanol fermentation facilities [24]. This endolysin derived from EcoSau was designated to LysKB317 in honor of Dr. Kenneth Bischoff. We demonstrated the effectiveness of exogenously added endolysin LysKB317 with predicted GH25 muramidase activity to a panel of Gram-positive bacterial species such as *Lactobacilli*. LysKB317 showed a robust antibacterial activity against eight species of *Lactobacillus*, including those that are problematic in the fuel ethanol industry. In addition, LysKB317 confirmed some activity against bacterial species such as *Acetobacter pomorum*, *Pediococcus spp*. *Streptococcus spp.*, and *Weissella confusa* isolated from commercial biorefineries. We determined the activity profile of LysKB317 under fermentation conditions, and with extended exposure to various pH, temperature, and percent ethanol. The robustness of LysKB317 was demonstrated in an experimentally infected corn mash fermentation to treat against *Lactobacillus fermentum* contamination and restored yield of ethanol fermentation by *S. cerevisiae*. Overall results showed the potential of endolysin LysKB317 as an alternative to conventional antibiotics to control contamination for the fuel ethanol industry.

## Results

### Lytic activity of purified endolysin LysKB317 confirmed

**Fig. 1:**
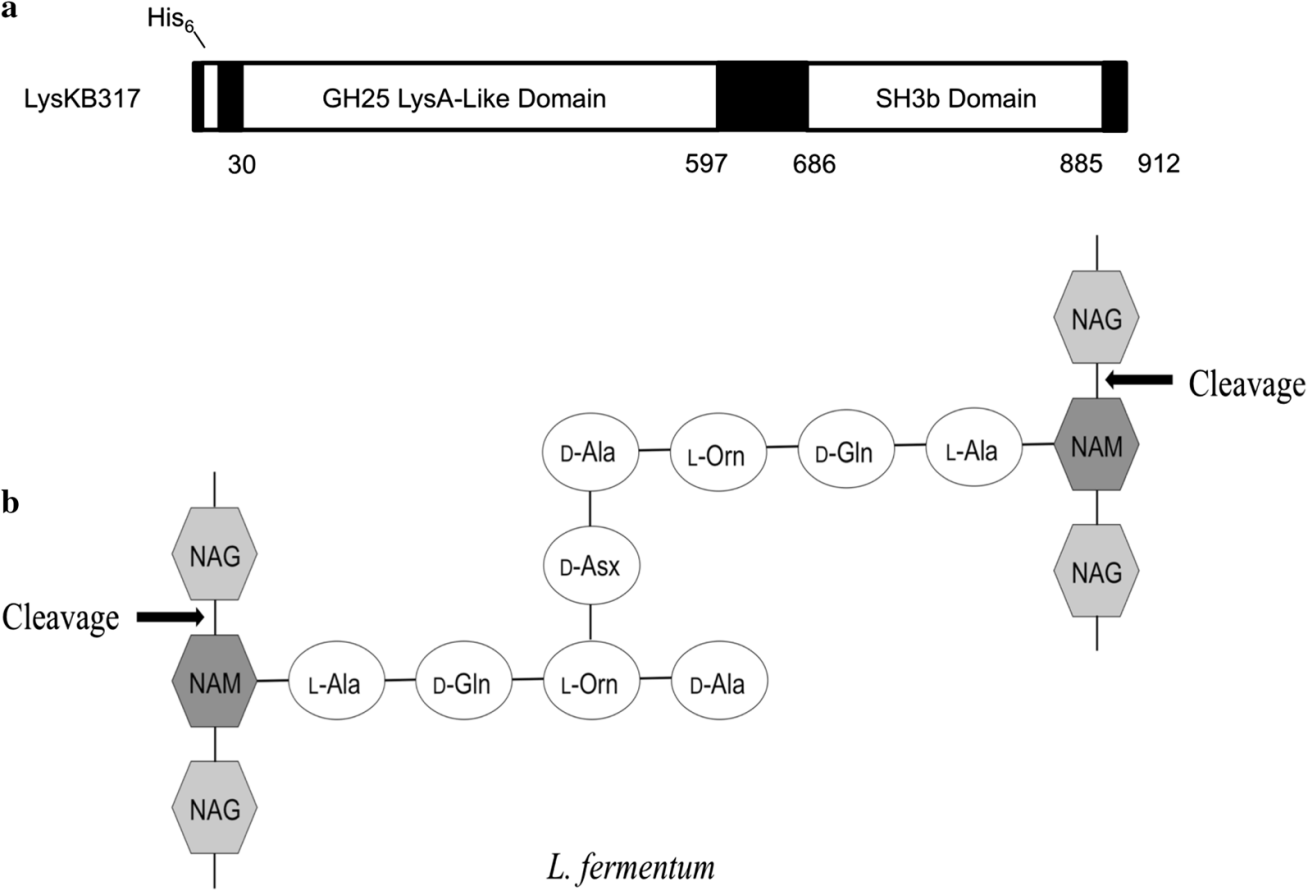
Schematic representation of LysKB317 domain structures and putative endolysin catalytic site on peptidoglycan of *L. fermentum*. **a** The LysKB317 is a recombinant (912 bp) 6 × His-tag phage lytic protein (33.8 kDa). The endolysin architecture (not to scale) consist of a fused N-terminal 6 × His-tag, glycosidase family 25 (GH25) lysin A-like enzymatic activity domain and a cell wall binding SH3b homologue domain based on amino acid homologies and conserved domains (catalytic and cell-wall binding) prediction data base. (GenBank accession number AIY32273.1). **b** The predicted LysKB317 catalytic site against *Lactobacillus fermentum* repeated peptidoglycan structure (modified from [4, 25, 26]) based on amino acid homologies to other biochemically characterized lytic enzymes [36]. D-Asx stands for D-Asp or amidated D-Asp (D-Asn [53])

**Fig. 2:**
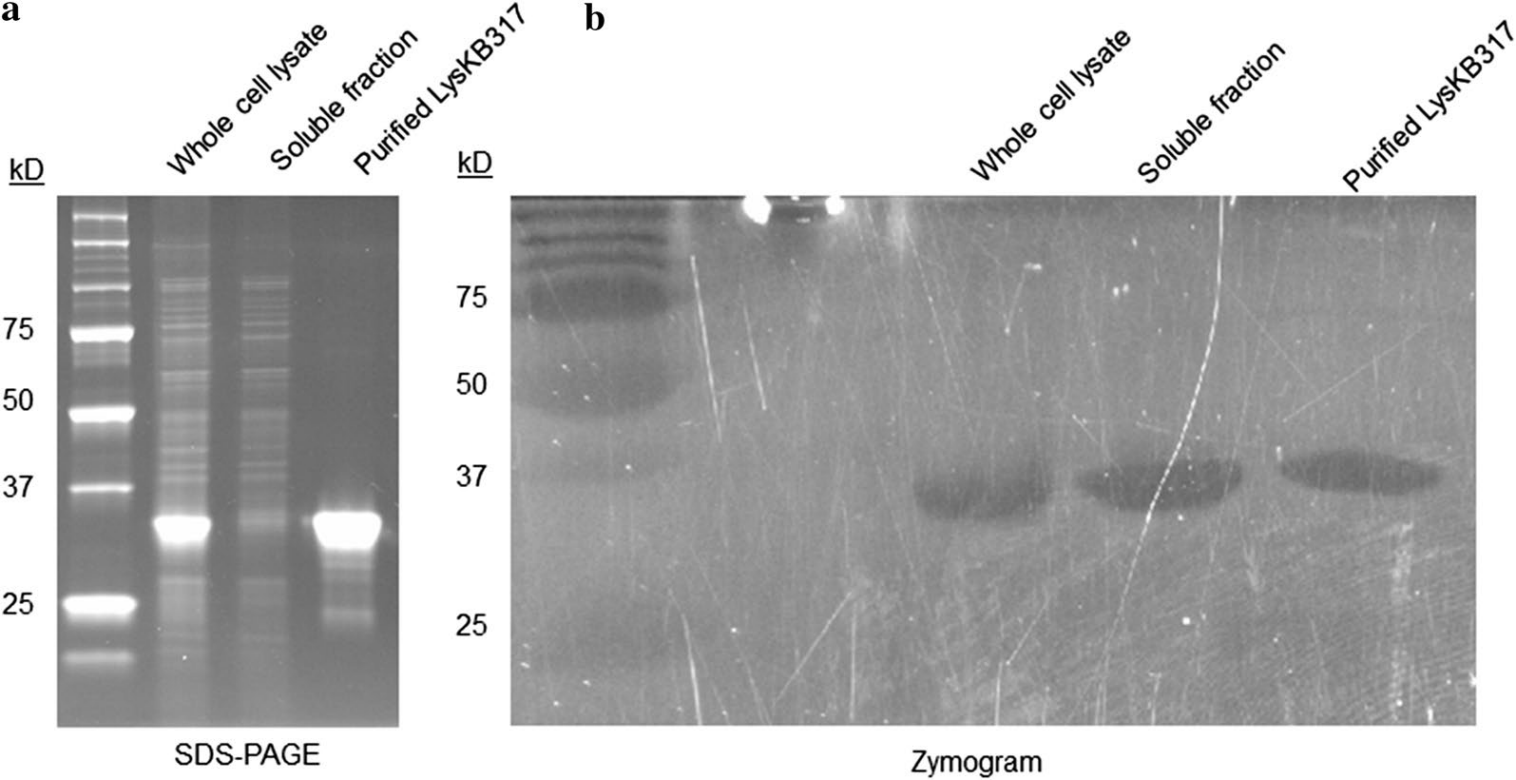
SDS-PAGE and recombinant LysKB317 endolysin zymogram assay. Whole cell lysate of overnight induced *E. coli* strain E. cloni 10G/pRham N-His Kan::LysKB317, soluble fraction from the whole cell lysate (2.5 µg) and purified LysKB317 endolysin (0.25 µg) were run on a 15% SDS–polyacrylamide gel. **a** Left panel: LabSafe Gel Blue stained gel. **b** Right panel: Zymogram activity assay. The gel contained bacterial cells of *L. fermentum* 0605-B44 polymerized within the gel matrix. Following electrophoresis, the gel was washed with deionized water for 1 h, then incubated in 1% Triton X-114, 50 mM Tris, pH 5.5 buffer until zones of clearing were visible (indicated hydrolase activity of LysKB317)

**Fig. 3:**
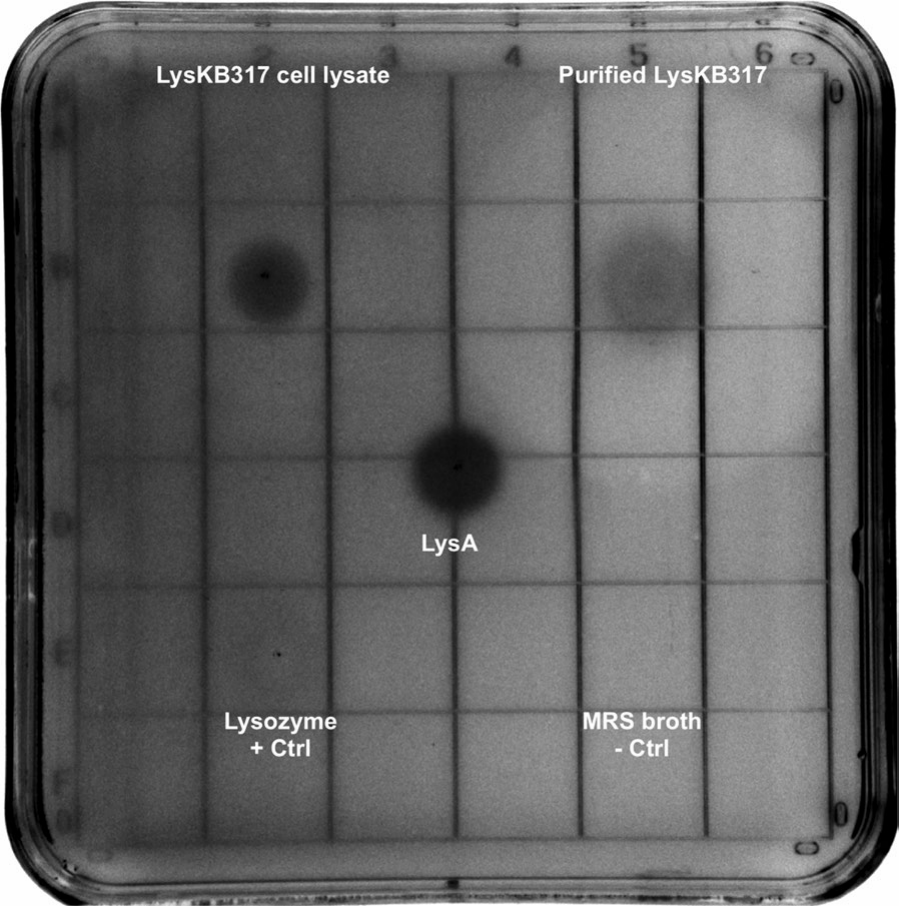
Spot plate assay of LysKB317 showed exolytic activity against *L. fermentum* 0315–25. Purified LysKB317 was spot onto an MRS agar plate that contained a soft top 0.7% agar with *L. fermentum* 0315–25 (OD_600_ = 0.8; 1 mL). Whole cell lysate expressing LysKB317 (5 µL; E. cloni 10G/pRham N-His kan::LysKB317; Table 1), purified LysA2 (5 µL; negative control; [54]), 20 µg/mL lysozyme (positive control), and 5 µL of MRS broth (negative control) were spotted on to plate and allow to air dry before incubating at 37 °C until zone of clearance can be visualized

We were able to express and purify the phage lytic protein in recombinant *E. coli* as the N-terminus 6 × His-tagged LysKB317 (Fig. 1a; GenBank accession number AIY32273.1). The endolysin consists of a glucohydrolase family 25 (GH25) muramidase-superfamily domain and a cell wall binding SH3b homologue domain (Fig. 1a). Based on blast search and homologous to protein sequences analyses with known functions, the predicted muramidase activity in LysKB317 is thought to cleave β-(1,4)-glycosidic bond of the peptidoglycan *N*-acetylglucosamine–*N*-acetylmuramic acid (NAG-NAM) linkages (Fig. 1b; [25, 26]) The SDS-PAGE and western blot analysis were performed on the nickel-NTA column purified protein, which produced a single prominent band for LysKB317 with the predicted molecular mass of 33.8 kDa (Fig. 2a and Additional file 1: Figure S1). Spot plate assay (using MRS agar plate incorporated 1 mL of live *L. fermentum* 0315–25 (OD_600_ = 0.8) in 0.7% soft top agar) demonstrated exolytic activity after spotting of 5 µL LysKB317 (either expressed whole cell lysate supernatant or purified LysKB317; Fig. 3). Under visual observation, the zone of clearing in both whole cell lysate supernatant or purified LysKB317 samples were significantly more pronounced compared to those of LysA (minimum activity against *L. fermentum* 0315–25; [4]), and lysozyme (positive control) confirming the exolytic activity of the enzyme. Zymogram analysis was performed with co-polymerized *L. fermentum* 0605-B44 into the gel matrix. Single translucent bands in the same size region as the predicted LysKB317 were clearly visible for whole cell lysate, soluble fraction, and purified enzyme of LysKB317 from the expression host *E. coli* (Fig. 2b).

### The LysKB317 lyses a range of *Lactobacillus* species

**Fig. 4:**
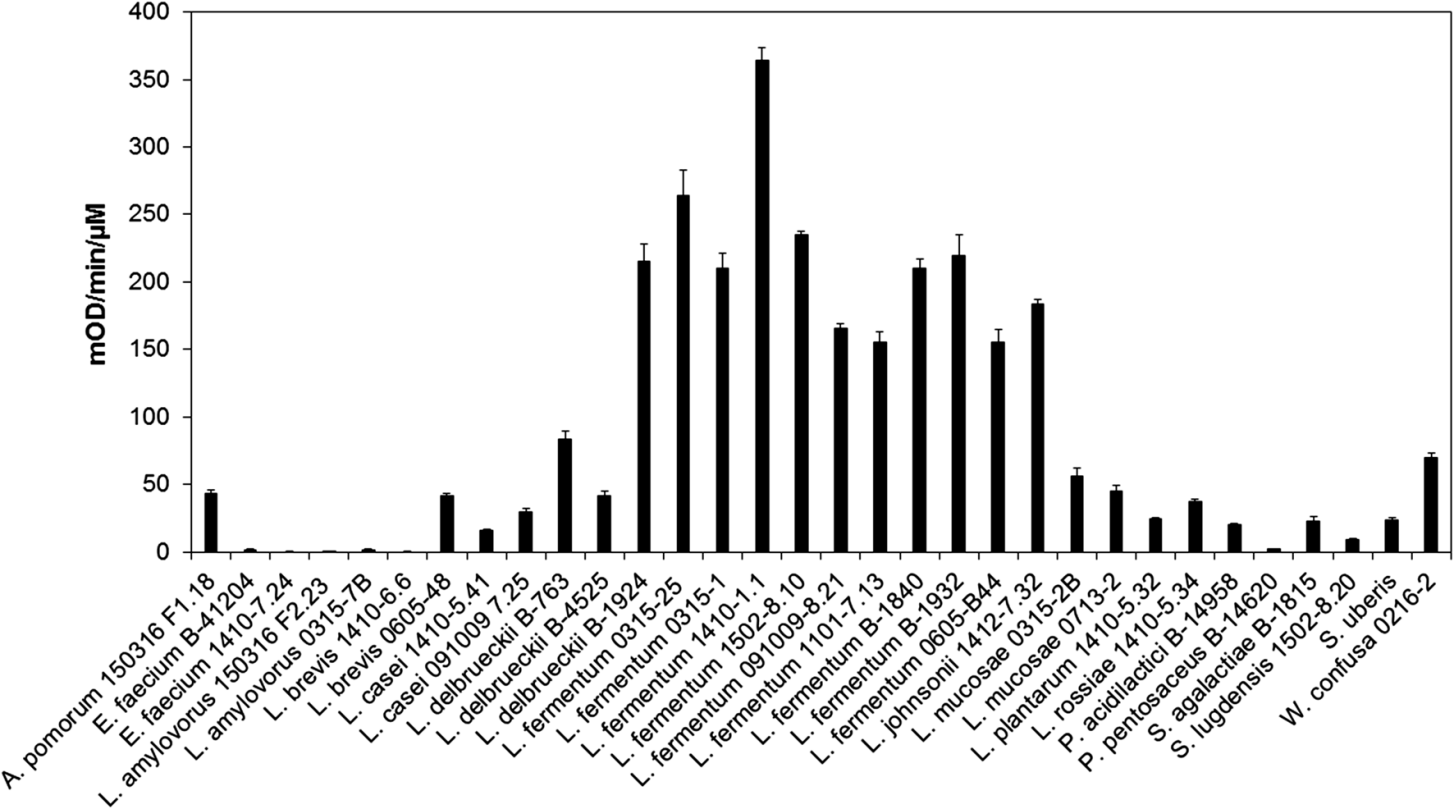
Activity of LysKB317 endolysin against various strains of bacteria isolated from fuel ethanol fermentation plant. Recombinant LysKB317 was tested for activity against the indicated strains isolated from ethanol fermentation facility using a turbidity reduction assay. In the wells of a 96-well microtiter plate, bacteria were suspended in 100 µL assay buffer (300 mM NaCl, 30% (v/v) glycerol, 21 mM citric acid, 58 mM Na_2_HPO_4_, pH5.5; OD_600 nm_ = 2.0), and 100 µL of enzyme reaction (1 µM) added. The change in optical density at 600 nm was measured over a period of 30 min. Data are reported as the mean change in O.D. per minute per µM enzyme (*n* = 3 independent replicates; error bars indicate standard errors of the mean (SEM))

Purified endolysin LysKB317 was tested using a turbidity reduction assay against several bacterial species (Table 1) isolated from commercial fuel ethanol fermentation plant (Fig. 4). LysKB317 had a strong lytic activity (> 100 OD_600_/min/µM enzyme; Fig. 4) against all of the *L.* f*ermentum* strains tested in the panel (Table 1). When other lactic acid bacteria species were included in the tests (Fig. 4), approximately 87% of the *Lactobacillus spp*. tested from the panel were susceptible to LysKB317 (Fig. 4). Furthermore, the endolysin showed lytic activity against bacterial species other than *Lactobacilli* such as *Weissella confuse*, *Acetobacter pomorum*, *Pediococcus acidilactici, Staphylococcus lugdunensis* and two species of *Streptococcus* (Fig. 4). However, bacterial species including *Enterococcus faecium, L. amylovorus*, and *L. brevis* presented minimum to none exolytic activity by the LysKB317.

**Table 1:** Bacteria and yeast strains used in this study

Bacteria and yeast	Relevant genotype/phenotype^a, b^	Reference or source^c^
*Escherichia coli*		
E. cloni 10G	*mcrA* Δ(*mrr*-*hsd*RMS-*mcr*BC) *end*A1 *rec*A1 φ80d*lac*ZΔM15 Δ*lac*X74 *ara*D139 Δ*(ara*,*leu*)7697 *gal*U *gal*K *rps*L (Str^R^) *nup*G λ^−^ *ton*A	Lucigen Co
E. cloni 10G/pUC57::LysKB317	Amp^R^, containing LysKB317 gene	GenScript, This study
E. cloni 10G/pRham N-His Kan::LysKB317	Kan^R^, containing LysKB317 gene	This study
BL21(DE3)	F^−^ *ompT hsdSB* (rB-mB-) *gal dcm* (DE3)	Invitrogen
BL21(DE3)/pET21a::LysA	Amp^R^, containing LysA gene	[4]
*Acetobacter pomorum*		
150316 F1.18	Wildtype	This study
*Enterococcus faecium*		
B-41204	Wildtype	NRRL
1410-7.24	Wildtype	NRRL
*Lactobacillus amylovorus*		
0315-7B	Wildtype	[51]
150316 F2.23	Wildtype	This Study
*Lactobacillus brevis*		
0605-48	Wildtype	[4]
1410-6.6	Wildtype	This study
*Lactobacillus casei*		
091009 7.25	Wildtype	This study
1410-5.41	Wildtype	This study
*Lactobacillus delbrueckii*		
B-1924	Wildtype	NRRL
B-4525	Wildtype	NRRL
B-763	Wildtype	NRRL
*Lactobacillus fermentum*		
B-1840	Wildtype	NRRL
B-1932	Wildtype	NRRL
0315-1	Wildtype	[51, 52]
0315-25	Wildtype	[4, 51]
0605-B44	Wildtype	[4]
091009–8.21	Wildtype	This study
1101-7.13	Wildtype	This study
1410-1.1	Wildtype	This study
1502-8.10	Wildtype	This study
*Lactobacillus johnsonii*		
1412-7.32	Wildtype	This study
*Lactobacillus mucosae*		
0713-2	Wildtype	[53]
0315-2B	Wildtype	This study
*Lactobacillus plantarum*		
1101 7.25	Wildtype	[52]
1410-5.32	Wildtype	This study
*Lactobacillus rossiae*		
1410-5.34	Wildtype	This study
*Pediococcus acidilactici*		
B-14958	Wildtype	NRRL
*Pediococcus pentosaceus*		
B-14620	Wildtype	NRRL
*Staphylococcus lugdunensis*		
1502-8.20	Wildtype	
*Streptococcus agalactiae*		
B-1815	Wildtype	NRRL
*Streptococcus uberis*		
		USDA
*Weissella confusa*		
0216-2	Wildtype	This study
*Saccharomyces cerevisiae*		
Y-2034		NRRL

^a^Amp^R^, Ampicillin resistant; Kan^R^, kanamycin resistant

^b^Wildtype microbial strains were isolated from a Midwestern dry-grind fuel ethanol plant and selected from a previous screen [15]

^c^USDA-ARS Culture Collection, Peoria, IL (also known as the NRRL Collection)

### LysKB317 endolysin remains active in the fermentation environment

**Fig. 5:**
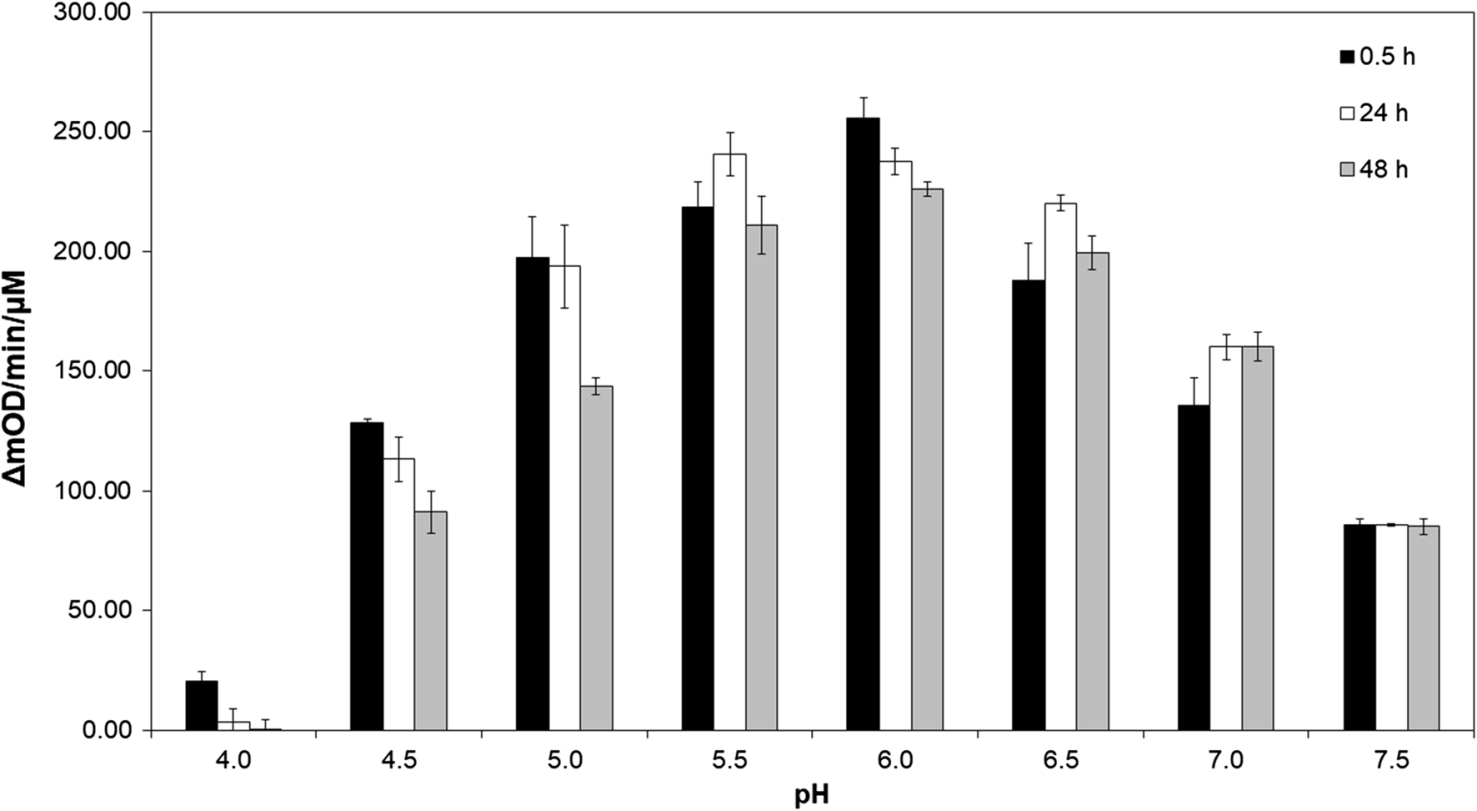
The endolysin LysKB317 pH stability profile over time under room temperature. Using the turbidity reduction assay, LysKB317 was tested in different pH (4.0–7.5) assay buffer and shown to be active at pH as low as 4.5 for 30 min. to pH 7.5 for 48 h. Time frames of 0.5 h (black bar), 24 h (white bar), and 48 h (gray bar) were arbitrary chosen to measure pH exposure over time. The change in optical density at 600 nm was measured over a period of 30 min at 37 °C. Data are reported as the mean change in O.D. per minute per µM enzyme (*n* = 3 independent replicates; error bars indicate standard errors of the mean (SEM))

**Fig. 6:**
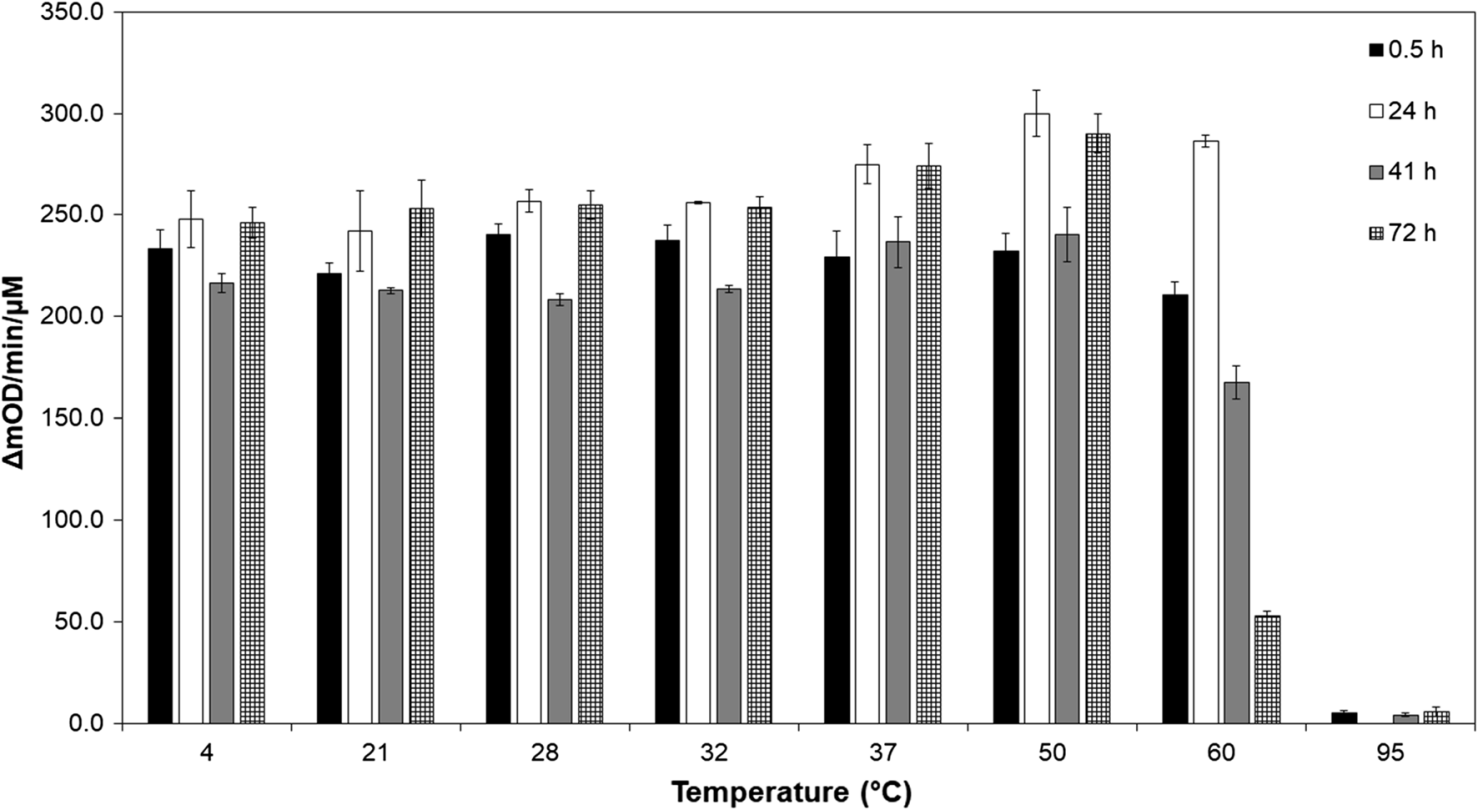
The endolysin LysKB317 can withstand high temperature of 60 °C up to 72 h. The temperature stability of the endolysin over time was measured using turbidity reduction assay. A temperature gradient ranging from 4 to 95 °C was tested over a period of 0.5 h (black bar), 24 h (white bar), 41 h (gray bar), and 72 h (checker bar). The change in optical density at 600 nm was measured over a period of 30 min. Data are reported as the mean change in O.D. per minute per µM enzyme (*n* = 3 independent replicates; error bars indicate standard errors of the mean (SEM))

**Fig. 7:**
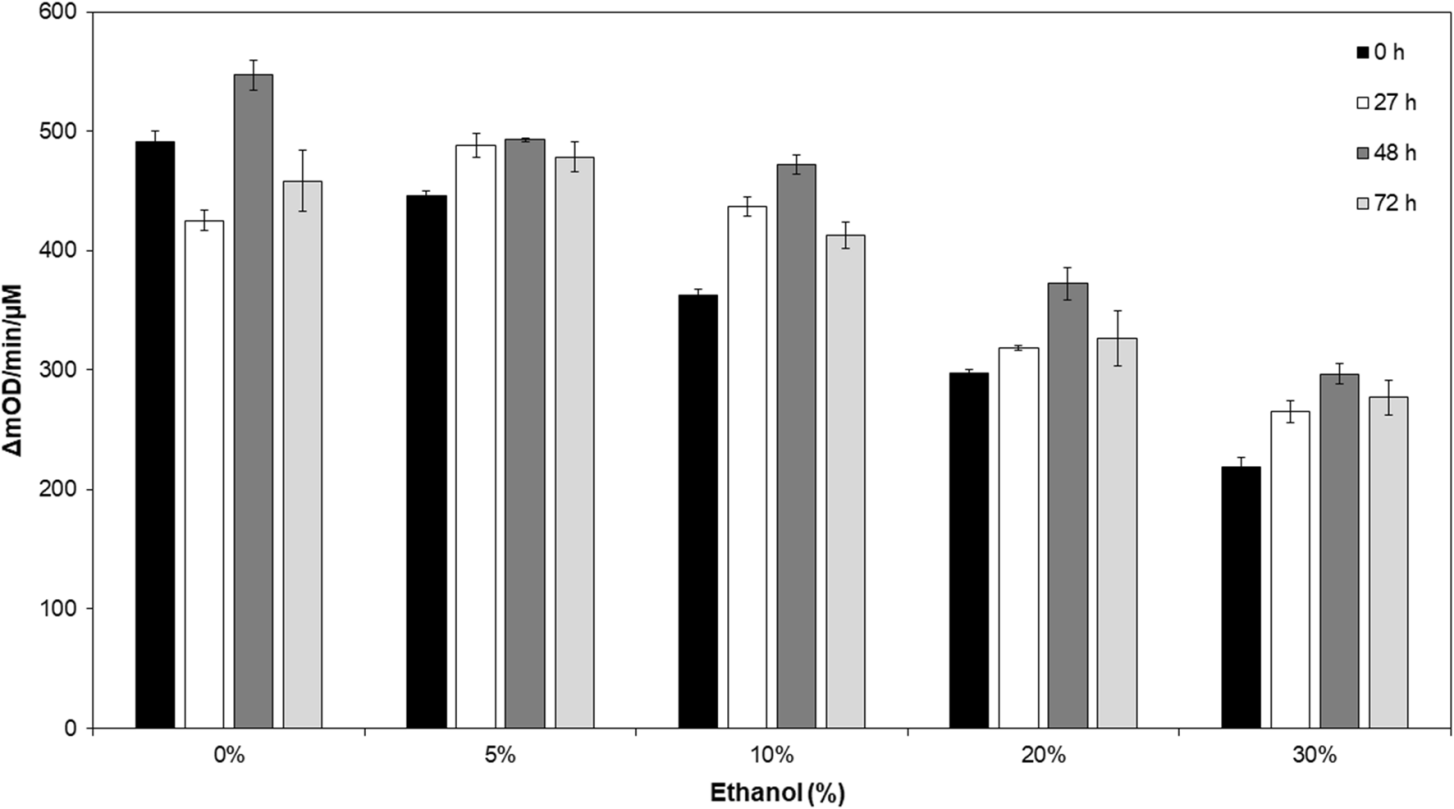
The endolysin LysKB317 is stable under 30% ethanol over 72 h at room temperature. A turbidity reduction assay was performed under various concentration of ethanol (0%, 5%, 10%, 20% and 30%) imitating conditions of LysKB317 would be exposed to in a fermentation tank at a bioethanol refinery plant. A time period of 0 h (black bar), 27 h (white bar), 48 h (dark gray bar), and 72 h (light gray bar) was used to test the stability of LysKB317 under ethanol exposure. The change in optical density at 600 nm was measured over a period of 30 min. Data are reported as the mean change in O.D. per minute per µM enzyme (*n* = 3 independent replicates; error bars indicate standard errors of the mean (SEM))

To examine the enzymatic activity of endolysin LysKB317 under typical fuel ethanol fermentation conditions, the enzyme was tested under a range of pH, temperature, and ethanol concentration over time using turbidity reduction assays. The optimal pH for LysKB317 was achieved at pH 6 and it was stable for up to at least 48 h (Fig. 5). In addition, the enzyme was functionally stable in a range of pH 4.5–7.5 up to at least 48 h, but the lytic activity of the endolysin at pH 4 was compromised. Thermostability of the enzyme was observed from 4 °C to 50 °C for at least 72 h (Fig. 6). At 60 °C, thermal stability of the enzyme started to deteriorate after 41 h of incubation and the lytic activity was abolished by 72 h. Minimal to no lytic activity was observed at 95 °C regardless of the time LysKB317 was incubated (Fig. 6). The presence of ethanol at or below 5%, did not have a significant impact on the lytic activity of the endolysin regardless of incubation time (0–72 h; Fig. 7). LysKB317 remained active upon exposure of ethanol concentration up to 30%, although activity was approximately 45–54% less than samples without added ethanol (Fig. 7).

### LysKB317 reduces *Lactobacillus* in a model fermentation flask

**Fig. 8:**
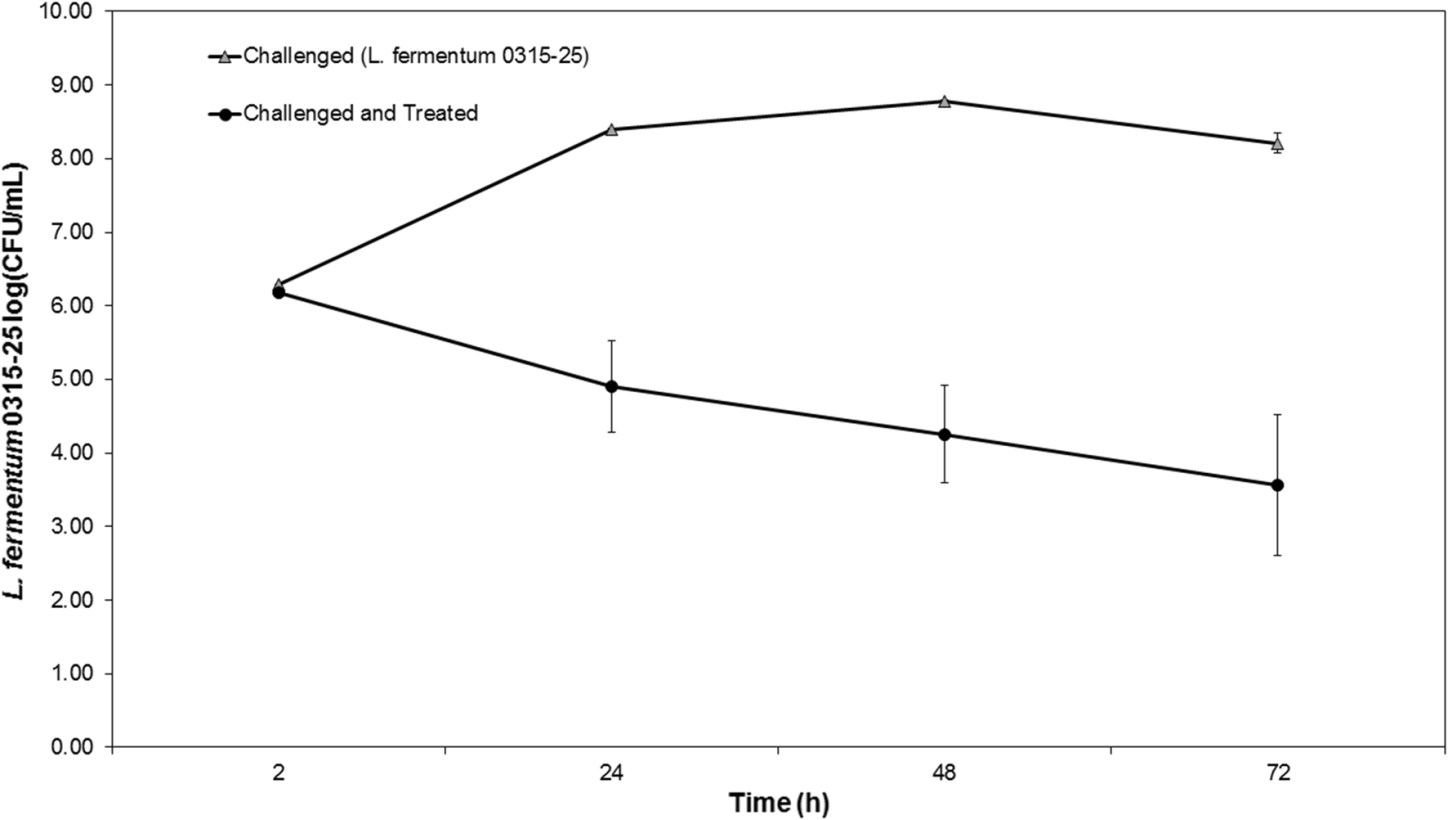
Small-scaled corn mash fermentation treatment with exogenously added LysKB317 reduced *L. fermentum* population. Cultures of *S. cerevisiae* grown on corn mash feedstock alone and exogenously added LysKB317 without *L. fermentum* challenge (served as negative controls not shown (below detection limit 3-log CFU/mL). Fermented corn mash challenged with 10^6^ CFU/mL of *L. fermentum* 0315–25 (gray triangle) served as positive control for contamination. Treatment applied to challenged fermentation corn mash (Black circle) showed significant bacterial contamination reduction over 72 h closely matching level of unchallenged fermented corn mash at 72 h. Data are reported in log CFU/mL growth of *L. fermentum.* Bacterial CFU counts were enumerated and log transformed every 24 h for 72 h using MRS agar plants. Error bars indicate standard errors of the mean (SEM); three independent replicates. *P < 0.05 based on one-way ANOVA

As described previously [4], we emulated fermentations using corn mash solids to test the effects on LysKB317 (Fig. 8). In experimentally infected corn mash fermentations, the addition of endolysin at 1 µM reduced bacterial load by approximately 3-log fold over time (black circle) compared to the challenged control fermentation (gray triangle), which rose above 9-log CFU/mL. Uninfected corn mash fermentations (negative control) and LysKB317-treated fermentations without infection (negative control) did not have detectable bacterial load over 3-log CFU/mL (limit of detection) were not included in the graph.

### Fermentation products of infected but LysKB317 treated were similar to those of uninfected controls

In our model bacterial infected flask yeast fermentation runs, the highest concentration of LysKB317 (10,000 nM ≡ 330 μg/mL) reduced bacterial load by 4-log fold CFU/mL change, while 100 nM (3.3 μg/mL) was able to reduce bacteria load by approximately 2-log fold (Table 2). The bacterial *L. fermentum* fermentation byproducts, such as lactic acid and acetic acid, which are known to inhibit *S. cerevisiae* and reduce ethanol yields were reduced significantly with the addition of LysKB317. Lactic acid was reduced more than 20% from 19.8 g/L to 15.4 g/L, while acetic acid decreased by more than 70% from 3.6 g/L to 1.0 g/L. The glucose utilization by *S. cerevisiae* after LysKB317 treatment (10,000 nM) in *L. fermentum* infected corn mash compared to uninfected corn mash had an over 98% improvement (from 38.9 g/mL glucose prior to treatment down to 0.7 g/mL glucose after endolysin treatment). End concentration of ethanol after fermentation had increased to 21.3 g/mL (~ 22% increase). The LysKB317-treated flask fermentation resulted comparable levels of glucose utilization and ethanol production when compared with that of uninfected flask fermentation controls.

**Table 2:** Treatment model for bacterial load and fermentation products of experimentally infected ethanol fermentations treated with exogenously added LysKB317 endolysin

Treatment	*L. fermentum* 0315–25				
LysKB317 (nM)	Log (CFU/mL)	Ethanol (g/L)	Glucose (g/L)	Lactic (g/L)	Acetic (g/L)
Control	< 3.0	117.4 ± 7.9	4.5 ± 2.6	15.2 ± 0.4	0.9 ± 0.2
0	8.4 ± 0.2	97.0 ± 7.5	38.9 ± 2.7	19.8 ± 0.5	3.6 ± 0.5
1	8.6 ± 0.1	94.6 ± 1.6	37.2 ± 0.6	19.9 ± 0.6	4.3 ± 0.8
10	8.5 ± 0.1	95.8 ± 7.4	26.1 ± 4.8	19.7 ± 0.1	2.7 ± 0.1
100	6.5 ± 0.2	113.1 ± 3.9	6.9 ± 4.1	16.3 ± 0.7	1.4 ± 0.3
1,000	6.4 ± 0.1	115.1 ± 13	2.1 ± 0.1	16.6 ± 0.1	1.3 ± 0.2
10,000	4.1 ± 1.4	118.3 ± 4.5	0.7 ± 0.3	15.4 ± 0.1	1.0 ± 0.3

Cultures of *S. cerevisiae* grown on corn mash feedstock were challenged with 10^6^ CFU/mL of *L. fermentum* 0315–25 [27], and treated with the indicated concentration of recombinant LysKB317 endolysin. The control culture was not challenged with *L. fermentum* 0315–25. After 72 h incubation, viable *L. fermentum* was determined by enumeration on MRS agar plates, and the fermentation broth was analyzed by HPLC for the following fermentation products: ethanol, residual glucose, lactic acid, and acetic acid

## Discussion

Bacterial contamination is inevitable during the propagation and fermentation processing of fuel ethanol production [12]. Mitigating bacterial contamination using antibiotics and adjustment of fermentation process, such as pH or temperature, have been used to control infection [23]. In addition, commercially available chemical-based products, such as hop acids and chlorine dioxide, have shown some success [27, 28]. However, there still is a need to improve the current technology by finding alternatives to control bacterial contamination in these types of biorefining processes. In the United States, ethanol production accounts for one of the largest industrial uses of antibiotics consumption [23, 29]. Prolonged excessive usage of antibiotics to treat bacterial contamination has raised concerns on the contribution to antimicrobial resistance [19, 21, 23]. Furthermore, it has been demonstrated that low concentration of biologically active antibiotic such as virginiamycin can persist in distilled grain coproducts when used in ethanol production facilities [30]. Low concentrations of bioactive antibiotics could potentially present a selection pressure resulting in anthropogenic influences that may contribute to bacterial resistance [31–33]. To date, rare accounts of resistance to bacterial peptidoglycan lytic enzymes have been reported, which makes it an effective and desirable alternative treatment to antibiotics [3, 34].

Our goal in this study was to demonstrate that purified endolysin LysKB317 could be a useful tool to mitigate bacterial contamination for the bioethanol industry [9, 14, 21]. The putative endolysin gene LysKB317 was first identified from EcoSau bacteriophage isolated from commercial sauerkraut [24]. The application of purified endolysin LysKB317 has demonstrated a high lytic activity against numerous Gram-positive LAB including several *Lactobacillus* species such as *L. fermentum*, which have previously been shown to negatively impact the rate of fermentation and often lead to stuck fermentations [19, 24, 35].

### Purified LysKB317 demonstrated lytic activity against most *Lactobacillus* species

Based on Pfam protein domain prediction, LysKB317 has a predicted peptidoglycan hydrolase similar to a glycoside hydrolase family 25 LysA-like domain active site (a muramidase) and a bacterial SH3b-like cell wall binding domain (Fig. 1a; [36]). A panel of 32 commonly found bacterial contaminant strains at ethanol fermentation facilities were tested (Table 1), and 26 (81% effective rate) strains were lysed by LysKB317. As a muramidase, LysKB317 is thought to cleave Gram-positive bacterial cell wall that shared similar peptidoglycan backbone. As a potential method to treat ethanol fermentation contaminants (e.g., *L. fermentum*), differences in the makeup of peptidoglycan chemotypes could have minimal impact on the catalytic activity of the endolysine to treat infection. Interestingly, in turbidity reduction assay, the highest (*L. fermentum*) and the lowest (*L. amylovorus*) lytic activities were all from *Lactobacillus* species (Fig**. **4). Not all peptidoglycan chemotypes of *Lactobacillus spp.* were equally sensitive to LysKB317 (Table 3; [37–41]) as seen with *L. fermentum* and *L. mucosae*. Differences in affinity of the SH3b cell wall binding domain and/or accessibility of the LysKB317 could affect target based on differences in strain specific cell wall surface moieties [42–45] cause interferences to the predicted cleavage site (Fig. 1b). More research is needed to determine the substrate specificity in *L. fermentum* by LysKB317 compared to *L. amylovorus*.

**Table 3:** Bacterial peptidoglycan chemotype in this study

Bacterial contaminants	Peptidoglycan chemotype^a,b^	References
*Acetobacter pomorum*	A3α L-Ala-D-meso-Dpm	[43]
*Enterococcus faecium*	A4α L-Lys-D-Asp	[39]
*Lactobacillus amylovorus*	A4α L-Lys-D-Asp	[28, 42]
*Lactobacillus brevis*	A4α L-Lys-D-Asp	[39]
*Lactobacillus casei*	A4α L-Lys-D-Asp	[39]
*Lactobacillus delbrueckii*	A4α L-Lys-D-Asp	[39]
*Lactobacillus fermentum*	A4β L-Orn-D-Asx	[39, 50]
*Lactobacillus johnsonii*	A4α L-Lys-D-Asp	[42]
*Lactobacillus mucosae*	A4β L-Orn-D-Asp	[42]
*Lactobacillus plantarum*	A1γ meso-Dpm-direct	[39]
*Lactobacillus rossiae*	N/A^b^	
*Pediococcus acidilactici*	A4α L-Lys-D-Asp	[39]
*Pediococcus pentosaceus*	A4α L-Lys-D-Asp	[42]
*Staphylococcus lugdunensis*	N/A^b^	
*Streptococcus agalactiae*	N/A^b^	
*Streptococcus uberis*	A3α L-Lys-L-Ala_2_	[42]
*Weissella confuse*	A3α L-Lys-L-Ala	[42]

^a^Dpm, 2,6-diaminopimelic acid; Orn, _L_-ornithine [28, 42]

^b^N/A, not available

### Endolysin LysKB317 exhibited a robust and stable characteristic under physical conditions of fermentation

Conditions typically found in fuel ethanol fermentation facility fermentation tank can have a temperature ranging from 30 to 35 °C (thermotolerant yeast strain 42–45 °C [46]) and pH range from pH 4 to 5.5 with an ethanol concentration no higher than 25% concentration for up to 48 h. The stability of the LysKB317 endolysin under such conditions is considered by us to be an effective alternative to antibiotics ([47]; Figs. 5, 6 and 7).

### LysKB317 is effective under small-scale corn mash fermentation conditions

Exogenous addition of purified LysKB317 alone was sufficient and successful in treating and controlling infected corn mash matrix (Fig. 8). Effective treatment seen in 50 mL Erlenmeyer flasks (Table 2) was encouraging in control *L. fermentum* bacterial load. The effectiveness in controlling bacterial contamination is also reflected upon the level of acetic and lactic acid in reducing the byproducts and restored ethanol production. Alternative methods to increase production of the lysin could be beneficial and will reduce cost at the industrial scale. Nevertheless, current method of exogenous addition of purified LysKB317 alone was able to control *L. fermentum* contamination and restore healthy fermentation characteristics.

## Conclusion

Bacteriophage-derived lytic endolysin enzyme such as LysKB317 is a strong candidate of antimicrobial control against LAB contamination in fuel ethanol fermentations. LysKB317 demonstrated the ability to lyse *L. fermentum* at pH, temperature, and ethanol concentrations similar to conditions found during fuel ethanol fermentations by at least two-log fold change in small-scale corn-mash fermentation. These qualities make LysKB317 an excellent candidate for antimicrobial control for use in biofuel fermentations.

## Methods

### Bacterial and yeast strains and culture conditions

Wildtype bacterial strains were isolated from a Midwestern dry-grind fuel ethanol plant and selected from a previous screen [15]. Unless otherwise stated, all bacterial strains described here (Table 1) were grown in its respective culture media. *Escherichia coli* strains in Miller’s LB (LB broth) medium (Difco Laboratories, Inc.). When used, ampicillin (Amp; Sigma-Aldrich, Inc.) at 100 µg/mL or kanamycin (Kan; Sigma-Aldrich, Inc.) at 50 µg/mL was added to LB media when required. Here we acknowledge newly reclassification and genera naming of some *Lactobacillus spp*. listed in this study (*e*.*g*., *Lactobacillus fermentum* as *Limosilactobacillus fermentum* and *Lactobacillus mucosae* as *Limosilactobacillus mucosae*) [48]. For consistency, older species names are being used here. *Lactobacillus *spp. and *Weissella.* were grown in Lactobacilli MRS (MRS broth) medium (Difco Laboratories, Inc.). *Acetobacter* and *Pediococcus* were grown in rapid lemonade spoilage organism broth (RLS broth; Sigma-Aldrich). *Enterococcus* strains were cultured in brain heart infusion broth (BHI; Bacto). *Streptococcus* were grown in tryptic soy broth (TSB; Difco Laboratories, Inc.). Unless otherwise stated, bacterial strains were inoculated at 37 °C with shaking (200 rpm), with expectation to *Lactobacillus spp.* (standstill incubation). *Saccharomyces cerevisiae* was grown in yeast extract peptone broth (YPD; BD Biosciences) at 32 °C with shaking (200 rpm).

### Construct, strains, and plasmids

Bacteriophage EcoSau endolysin gene (LysKB317; GenBank accession number KP027015.1; protein accession number AIY32273.1 [24]) was codon optimized for *E. coli* expression and synthesized by GenScript (Table 4). Plasmid pUC57 carrying LysKB317 was transformed into *E. coli* (E. cloni 10G; Lucigen Co.) for plasmid propagation (Table 1). Primer set Sau_F and Sau_R (Table 5) was used to amplify the 894 bp LysKB317 gene insert. PCR amplicon was cleaned using QIAquick PCR purification kit (Qiagen) and cloned into pRham N-His Kan vector (Table 4) using *E. coli* strain E. cloni 10G (Lucigen Co., Table 1) per manufacture protocol. The LysKB317 plasmid construct was Sanger sequenced verified using primer set pRham_F and pETite_R (Table 5).

**Table 4:** Plasmids used in this study

Plasmid	Relevant genotype^a^	Reference
pUC57::LysKB317	Amp^R^, containing LysKB317 gene fragment	GenScript
pRham N-His Kan	Kan^R^, Expresso rhamnose cloning vector	Lucigen Co
pRham N-His Kan::LysKB317	Kan^R^, containing the LysKB317	This study

**Table 5:** Primers used in this study

Primer name	Sequence (5′–3′)	Purpose	Reference
Sau_F	CATCATCACCACCATCACGCACTTTACGTAGTTGACGTT	Amplification of LysKB317 for cloning	This study
Sau_R	GTGGCGGCCGCTCTATTATTTAAAGGTTCCGAATGCTTC		
pRham_F	GCTTTTTAGACTGGTCGTAGGGAG	Verify gene insert	Lucigen Co
pETite_R	CTCAAGACCCGTTTAGAGGC		

### Expression and purification of LysKB317

Commercially available Gram-negative *E. coli* Expresso SUMO protein expression system (Lucigen) was used to express Gram-positive *Lactobacilli* toxin. The LysKB317 endolysin protein was over expressed in *E. coli* (E. cloni 10G/pRham N-His Kan::LysKB317; Table 1) via 0.2% (w/v) L-rhamnose (Sigma) induction in 1 L LB broth with Kan at 37 °C shaking (200 rpm) overnight. Cells were harvested by 4 °C centrifugation at 5,000 × *g* for 20 min. Cells were then lysed with B-PER (Thermo Scientific) and the addition of freshly prepared lysozyme (20 mg/mL in 1 mM Tris–HCl, pH8.0; Thermo Scientific), DNaseI (10 U/mL; Thermo Scientific), and RNase I (10 U/mL; Thermo Scientific), followed by gentle inversion for 20 min at room temperature. Soluble protein fraction was separated from whole cell lysate via 15,000×*g* centrifugation at 4 °C for 5 min and purified using HisPur Ni–NTA Superflow Agarose (Thermo Scientific). Nickel resin was washed with 40 column volumes (CV) of lysis buffer, and 15 CV of wash buffer (50 mM NaH_2_PO_4_, 300 mM NaCl, 20 mM imidazole, and 30% glycerol, pH 8.0). Bound LysKB317 His_6_-tagged protein was eluted with elution buffer (50 mM NaH_2_PO_4_, 300 mM NaCl, 250 mM imidazole, and 30% glycerol, pH 8.0) and filter sterilized with 0.22 µm. Concentration of protein was determined using a Qubit 3 fluorometer (Thermo Fisher Scientific) and Qubit Protein Assay Kit (Thermo Fisher Scientific). A sodium dodecyl sulfate (SDS)–polyacrylamide gel electrophoresis (PAGE) was used to determine the protein purities. Purified N-terminus His-tagged LysKB317 recombinant protein was resuspended in 1 × Laemmli sample buffer (Bio-Rad laboratories, Inc.) and boiled for 5–10 min. Fifteen microliters of the boiled sample and protein standard (Precision Plus Protein All Blue standard; Bio-Rad) were loaded side-by-side onto an Any kD Tris–glycine precast gel (Bio-Rad) for (SDS-PAGE) protein separation at 100 V for 70 min. The gel was stained with LabSafe Gel Blue stain (G-Biosciences) for 1 h at room temperature with gentle agitation, and then destained with deionized (DI) water for at least 1 h.

### Expression and purification of endolysin LysA

The endolysin LysA (36.4 kD; glycosidase) known to inhibit *Lactobacilli* spp. was chosen as an endolysin comparison to LysKB317 [4]. *E. coli* BL21(DE3)/pET21a::LysA (Table 1) was induced similar to previously discussed methods [49] with 0.5 mM of isopropyl β-D-1-thiogalactopyranoside (IPTG; Sigma) in LB broth and ampicillin (100 µg/mL) overnight at 37 °C with agitation. LysA endolysin was purified using methods described above.

### Western blot analysis

Purified *N*-terminus His-tagged LysKB317 recombinant protein described above was separated by SDS-PAGE as previously described. A Trans-Blot turbo transfer system (Bio-Rad) was used for protein transfer onto a low-fluorescence polyvinylidene difluoride (PVDF) membrane with 0.2 µm pore size (Bio-Rad). Protein electrophoresis transfer was verified using Ponceau S staining (Cell Signaling Technology, Inc.). Nonspecific binding was blocked by 3% bovine serum albumin (BSA) in 1 × tris-buffered saline (TBS) containing 0.1% Tween-20 (Sigma-Aldrich). Mouse anti-His-tag antibody conjugated to DyLight 488 was applied and incubated at 4 °C overnight (1:1,000; Thermo Fisher Scientific). Fluorescent band signals were detected using a ChemiDoc XRS + imaging system (Bio-Rad).

### Spot plate assay

Bacterial strains (Table 1) were inoculated in 5 mL MRS at 37 °C without shaking and grown to OD_600 nm_ of approximately 0.8. Bacterial strains (1 mL) were then mixed with 0.7% agar (50 °C), and 0.5 mL of plate buffer (50 mM NaH_2_PO_4_, pH 7.0), then poured onto pre-solidified regular MRS agar plate (1.6%) and allowed to air dry. Purified LysKB317 protein at a pre-determined concentration [1.0 µM; 5 µL] was pipetted onto the MRS agar and dried for 10 – 15 min. Sterile water was used as negative control. The plate was then incubated at 37 °C overnight. Strains that exhibited zones of clearance were deemed susceptible to LysKB317. As controls, 5 µL of MRS broth served as the negative control and 5 µL of 20 µg/mL purified endolysin LysA and lysozyme separately served as the positive control [50].

### Zymogram

Zymogram analysis was performed based on a previously described method with slight modification [4]. Briefly, *L. fermentum* 0315–25 cells (Table 1) were grown to mid-log phase in 50 mL MRS media and pelleted at 4,000×*g* for 15 min. Cells were washed with 10 mL of zymogram buffer (10 mM Tris, 150 mM NaCl, pH 7.5), harvested and resuspended in 300 µL zymogram buffer resulting in a final volume of approximately 600 µL. The purified LysKB317 protein described above, and protein standard (Precision Plus Protein All Blue standard; Bio-Rad) were run in parallel in two separate 15% SDS-PAGE gels. One gel contained 600 µL of resuspended *L. fermentum* 0315–25 cell (zymogram), and the other gel contained only 600 µL of buffer (negative control). Each of which was added prior to gel polymerization. Gels were electrophoresed for 1–2 h at 150 V until completion. SDS-PAGE gels were stained using LabSafe GEL Blue (G-Biosciences) and washed in deionized (DI) water for 1 h at room temperature. Additional de-staining incubation was done with gels submerged in de-staining buffer (50 mM Tris–HCl, 1% Triton X-114, pH 5.5) at room temperature with gentle swirling overnight or until translucent bands is clearly visible as described [49].

### Turbidity reduction assay

Turbidity reduction assay was performed at 37 °C, unless otherwise stated, in Synergy 2 Microplate Reader (BioTek Inc.) with purified LysKB317 protein (described above) diluted in turbidity reduction assay buffer (300 mM NaCl, 30% (v/v) glycerol, 21 mM citric acid, 58 mM NaH_2_PO_4_, pH 5.5) to 1 μM concentration. *Lactobacillus* cultures (listed in Table 1) used in the turbidity reduction assay were prepared as previously described [4]. Briefly, bacterial cells were inoculated in 50 mL MRS media and grown to mid-log phase. Cells were washed in phosphate-buffered saline (PBS; pH 7.4, 30% glycerol) before being adjusted to an optical density (OD_600 nm_) = 2.0. Aliquots of 1 mL of cells were then centrifuged and pellet resuspended in 1 mL of turbidity reduction assay buffer. Each of the designated experimental wells of a 96-well microtiter plate (flat bottom; Falcon) contained 100 μL bacterial suspension and 100 μL of 1 μM endolysin. Wells containing bacterial cell suspension (100 μL) without endolysin (100 μL turbidity reduction assay buffer) were used to control the rate of autolysis of bacterial cells. Immediately upon addition of endolysin to bacterial suspension, absorbance readings (OD_600_) were recorded every 30 s for 30 min. Treatment and control wells were run in triplicates. Specific actives were determined by (ΔmOD_600 nm_/min/µM) described by Becker et al. [51].

### Temperature, pH and ethanol sensitivity assays

Thermostability of LysKB317 was determined by placing 1 µM of purified endolysin in turbidity reduction assay buffer at each pre-determined temperature (4°, 21°, 28°, 32°, 37°, 50°, 60°, and 95 °C) and incubated for 0.5, 24, 41, and 72 h before performing the turbidity reduction assay described above at 37 °C. In a similar fashion, 1 µM of purified LysKB317 was added to pre-determined turbidity reduction assay buffer at pH 4.0, 4.5, 5.0, 5.5, 6.0, 6.5, 7.0, and 7.5 (21 mM citric acid, 58 mM NaH_2_PO_4_ buffer adjusted to the pH indicated) for 0.5, 24, and 48 h at room temperature prior to performing the turbidity reduction assay as described above for 30 min at 37 °C. Ethanol from 0–30% (g/100 ml) concentrations was added to the LysKB317 buffer for pre-determined amount of time (0–72 h) before performing the turbidity reduction assay.

### Preparation of small-scale corn mash fermentation

This was done as described in Bischoff et al. and Roach et al. [4, 35]. Briefly, the *S. cerevisiae* strain NRRL Y-2034 (Table 1) was grown overnight in YP broth supplemented with 5% (w/v) glucose at 32 °C with 200 rpm shaking. The infection *L. fermentum* strain 0315–25 (Table 1) was grown in static MRS media at 37 °C to mid-log phase (OD_600_ nm = 0.4–0.6). Both yeast and bacteria cells were collected via centrifugation and inocula were resuspended in sterile phosphate-buffered saline (PBS; pH 7.4, Fisher Scientific) to OD_600_ nm equivalent of 80 for yeast, and OD_600_ nm equivalent of 8.0 for *L. fermentum* 0315–25. One OD_600_ nm is approximately 6 × 10^7^ CFU/mL for yeast and 1 × 10^8^ CFU/mL for bacteria. Corn mash (approximately 33% solids) was collected from a commercial dry-grind ethanol facility and stored at − 20 °C. Verification of aliquots of corn mash samples onto MRS agar did not detect transient bacteria in the mash (< 10^2^ CFU/mL). In separate 50 mL Erlenmeyer flasks, 40 mL corn mash with ammonium sulfate (0.12%, w/v) and glucoamylase (20 μL of Optidex L-500; Genecor International Inc.) were dispensed.

Purified endolysin LysKB317 (1 µM) was added with 0.5 mL *S. cerevisiae* inoculum and when indicated, 0.5 mL challenged bacterial inoculum was added sequentially at time 0. Each flask was plugged with a rubber stopper containing a 20-gauge 0.9 mm × 40 mm PrecisionGlide needle (Becton Dickinson) to vent excess CO_2_. Flasks were initially incubated at 32 °C with 100 rpm shaking for 3 h to acclimate yeast. All fermentation flasks were briefly removed from the incubator prior to the beginning of the experiment. Designated flasks were then seeded with 0.5 mL of *L. fermentum* with half of the flasks getting endolysin treatment before all flasks were returned to the incubator (32 °C and 100 rpm shaking). Two-hundred fifty microliters of samples were taken at 0, 0.5, 1.0, 1.5, 4, 48, and 72 h and diluted in PBS (pH 7.4, 1:10). Time course up to 72 h was chosen as most fermentation without bacterial contamination has been shown to be completed by 72 h [52]. Fermentation samples were tittered for bacterial counts on 1.5% MRS agar and yeast inhibitor (100 µg/mL; cycloheximide) by serial dilution plating using the Eddy Jet 2 spiral plater (IUL Instruments) set in the E mode 50 (50 µL sample). Plates were then incubated anaerobically using the Anaero Pack System (Mitsubishi) at 37 °C for 18 h [49]. Colony forming unit/mL (CFU/mL) were numerated using a Flash & Go plate reader (IUL Instruments) with ≥ 10 CFU minimum detection limit at 3.3 log_10_ (CFU/mL). Based on unpublished data, LysKB317 does not show any detectable inhibitory effect against *S. cerevisiae*, thus no yeast cell counts were collected. As previously described, a high-performance liquid chromatography (HPLC) system with 300 mm Aminex HPX 87H column (Bio-Rad laboratories, Inc.) was used to quantify presence of acetic acid, galactose, glucose, and lactic acid [35].

### Statistical analysis

Where appropriate, experimental results were analyzed using one-way analysis of variance (ANOVA) test (Microsoft Excel 2019).

## Supplementary information

**Additional file 1.** Additional figures.

## Data Availability

Data and material will be available on Ag Data Commons at https://www.data.nal.usda.gov

## Abbreviations

ANOVA: Analysis of variance
BHI: Brain heart infusion broth
BSA: Bovine serum albumin
CFU: Colony forming unit
HPLC: High-performance liquid chromatography
IPTG: Isopropyl-β-D-1-thiogalactopyranoside
LAB: Lactic acid bacteria
LB: Luria–Bertani
MRS: De man, rogosa and sharp
OD: Optical density
PBS: Phosphate-buffered saline
PVDF: Polyvinylidene difluoride membrane
RLS: Rapid lemonade spoilage organism broth
SDS-PAGE: Sodium dodecyl sulfate–polyacrylamide gel electrophoresis
SEM: Standard errors of the mean
TBS: Tris-buffered saline
TSB: Tryptic soy broth
YP: Yeast extract peptone
