# Supplementary material for: Recombinant bacteriophage LysKB317 endolysin mitigates Lactobacillus infection of corn mash fermentations
Source: Biotechnol Biofuels. 2020 Sep 8;13:157. doi: 10.1186/s13068-020-01795-9 (PMC7488000; doi:10.1186/s13068-020-01795-9)

**Recombinant Bacteriophage LysKB317 Endolysin Mitigates *Lactobacillus* Infection of Corn Mash Fermentations**

Shao-Yeh Lu^1^, Kenneth M. Bischoff^1^, Joseph O. Rich^1,2^, Siqing Liu^1^, and Christopher D. Skory^1*^

^1^Renewable Product Technology Research Unit, National Center for Agricultural Utilization Research, Agricultural Research Service, U.S. Department of Agriculture, Peoria, IL 61604, USA

^2^Agricultural Research Service, U.S. Department of Agriculture, Fort Collins, CO, 80526, USA

*Corresponding author:

Chris D. Skory, Ph.D., USDA-ARS-NCAUR, 1815 North University Street, Peoria, IL 61604-3902, USA; (309) 681-6375; chris.skory@usda.gov

Running Title: LysKB317 endolysin mitigates *Lactobacillus* contamination

Keywords: Phage endolysin, contamination, fuel ethanol, *Lactobacillus*, antimicrobial.

**Additional Materials**

**Figure S1. Endolysin LysKB317 protein purification.** Endolysin LysKB317 was induced with 0.2% L-rhamnose overnight at 37°C with agitation. Fifteen micro-liter of samples from *E. coli*/pRham N-His Kan::LysKB317 at each protein purification process were taken (whole cell lysate, insoluble fraction, soluble fraction, sample flow through, wash steps and protein elution). Samples were boiled for 10 min in sample buffer and loaded 5 µL sample per well into a precast 8 – 16% sodium dodecyl sulfate polyacrylamide gel and performed electrophoresis (SDS-PAGE; Left panel). Western blot was performed by transferring protein samples onto polyvinylidene difluoride (PVDF) membrane and probed with anti-6His antibody detecting the presence of LysKB317 (Right panel).


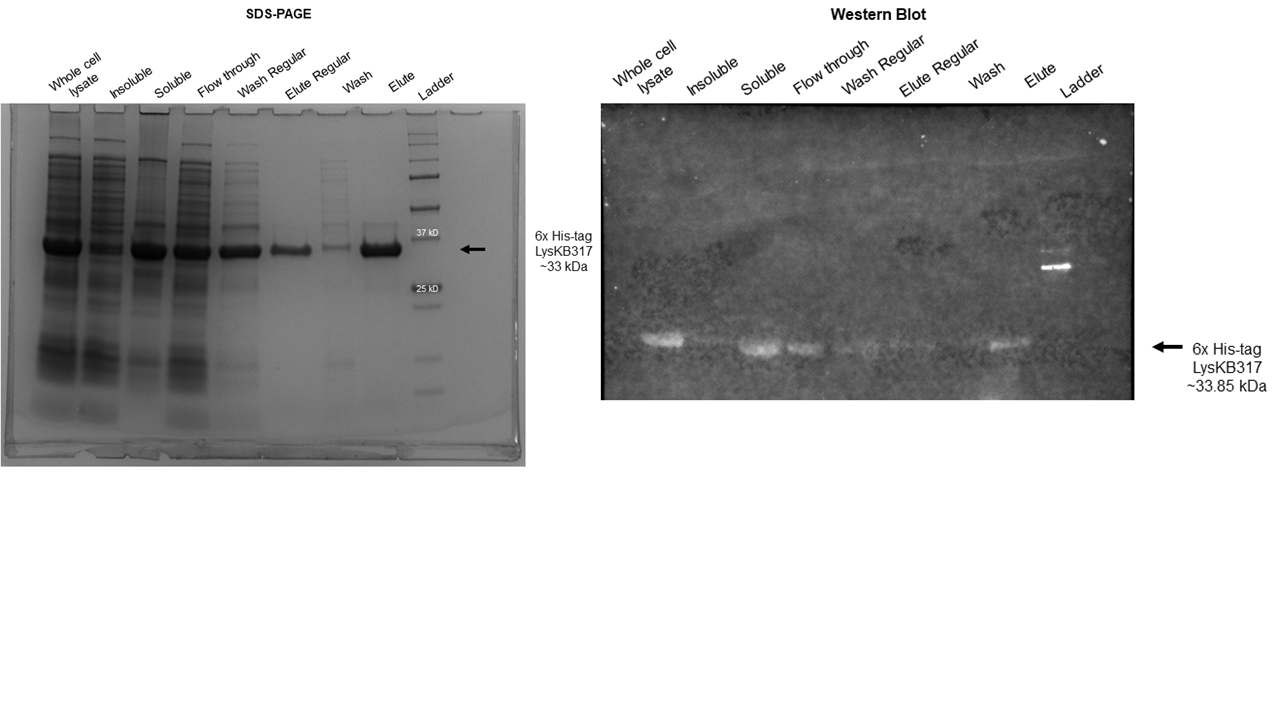


**Figure S2. Endolysin LysA protein expression.** Endolysin LysA was induced with 0.5 mM IPTG overnight at 37°C with 200 rpm shaking. Fifteen micro-liter of eluted protein samples from *E. coli* BL21(DE3)/pET21a::LysA were taken. Samples [(whole cell lysate and purified enzyme LysA (36.4 kDa); LysKB317 (33 kDa)] were boiled for 10 min in 15 µL sample buffer and loaded 5 µL sample per well into a precast 8 – 16% sodium dodecyl sulfate polyacrylamide gel and performed electrophoresis (SDS-PAGE; Left panel). Western blot was performed by transferring protein samples onto polyvinylidene difluoride (PVDF) membrane and probed with anti-6His antibody detecting the presence of LysA and LysKB317 (Right panel).


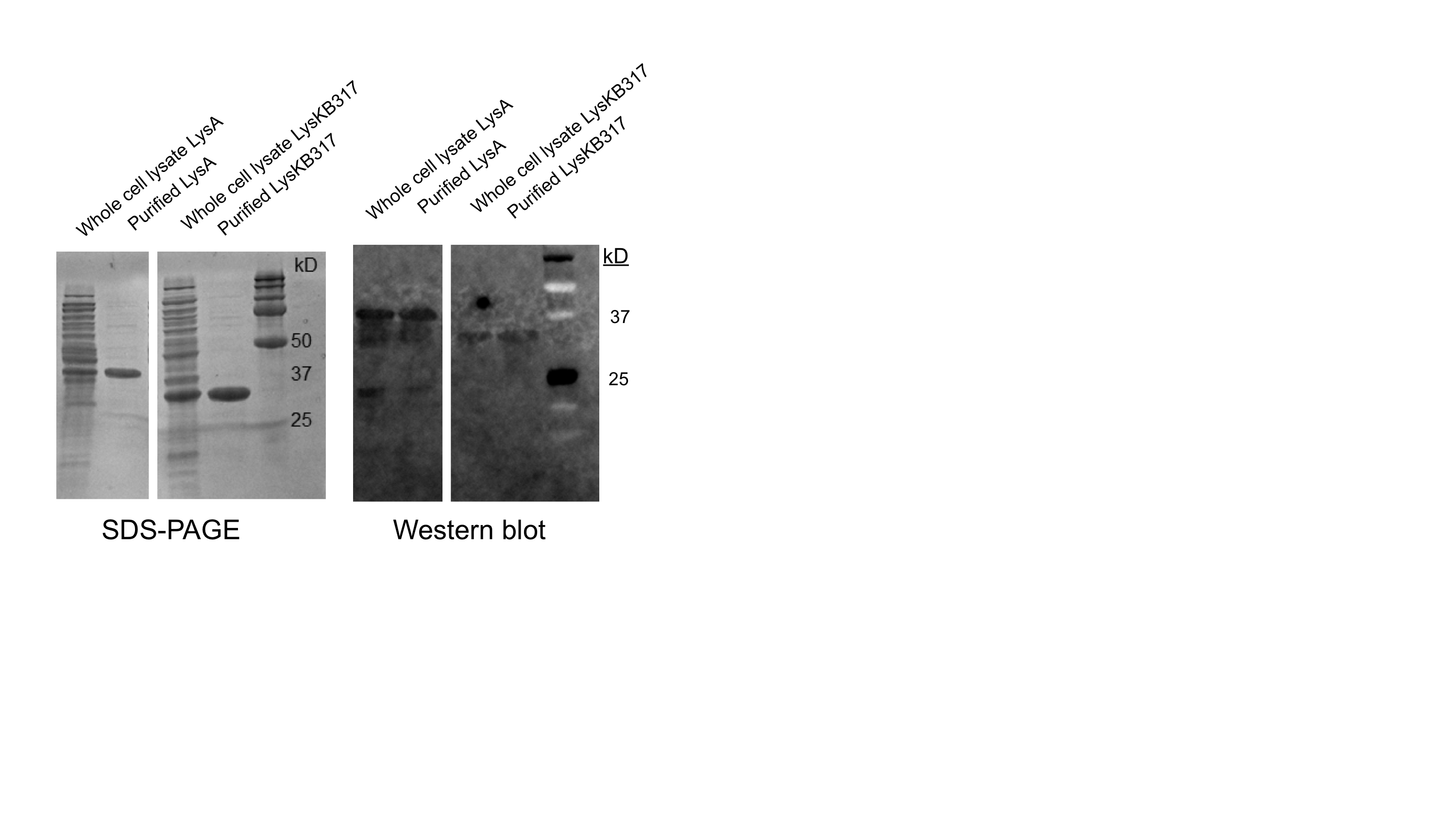

Supplement: Supplementary file 1 — Additional file 1. Additional figures. [file 13068_2020_1795_MOESM1_ESM.docx]
